# Role of Fermented Goat Milk on Liver Gene and Protein Profiles Related to Iron Metabolism during Anemia Recovery

**DOI:** 10.3390/nu12051336

**Published:** 2020-05-08

**Authors:** Jorge Moreno-Fernandez, María J. M. Alférez, Inmaculada López-Aliaga, Javier Díaz-Castro

**Affiliations:** 1 Department of Physiology, University of Granada, 18071 Granada, Spain; jorgemf@ugr.es (J.M.-F.); malferez@ugr.es (M.J.M.A.); javierdc@ugr.es (J.D.-C.); 2 Institute of Nutrition and Food Technology “José Mataix Verdú”, University of Granada, 18071 Granada, Spain; 3 Nutrition and Food Sciences Ph.D. Program, University of Granada, 18071 Granada, Spain

**Keywords:** fermented cow and goat milk, anemia, iron homeostasis, iron repletion, gene and protein expression

## Abstract

Despite the crucial role of the liver as the central regulator of iron homeostasis, no studies have directly tested the modulation of liver gene and protein expression patterns during iron deficiency instauration and recovery with fermented milks. Fermented goat milk consumption improves the key proteins of intestinal iron metabolism during iron deficiency recovery, enhancing the digestive and metabolic utilization of iron. The aim of this study was to assess the influence of fermented goat or cow milk consumption on liver iron homeostasis during iron-deficiency anemia recovery with normal or iron-overload diets. Analysis included iron status biomarkers, gene and protein expression in hepatocytes. In general, fermented goat milk consumption either with normal or high iron content up-regulated liver DMT1, FPN1 and FTL1 gene expression and DMT1 and FPN1 protein expression. However, HAMP mRNA expression was lower in all groups of animals fed fermented goat milk. Additionally, hepcidin protein expression decreased in control and anemic animals fed fermented goat milk with normal iron content. In conclusion, fermented goat milk potentiates the up-regulation of key genes coding for proteins involved in iron metabolism, such as DMT1, and FPN1, FTL1 and down-regulation of HAMP, playing a key role in enhanced iron repletion during anemia recovery, inducing a physiological adaptation of the liver key genes and proteins coordinated with the fluctuation of the cellular iron levels, favoring whole-body iron homeostasis.

## 1. Introduction

Iron deficiency anemia (IDA) is a highly prevalent pathology and a medical condition in the clinical practice, affecting more than two billion people worldwide. This public health problem has a negative impact on the lives of infants and fertile women worldwide [1]. Daily, in the duodenum and proximal jejunum, 2 mg of iron is absorbed. The non-heme iron in the diet is in Fe^3+^ form and it must be reduced to ferrous form before it can be absorbed by the action of duodenal cytochrome B. Fe^2+^ is then transported across the apical membrane of enterocytes by the divalent metal transporter 1 (DMT1). This protein transports some divalent cations including ferrous iron. Iron crosses the basolateral membrane of intestinal enterocytes by the action of ferroportin, an iron exporter, entering into systemic circulation. After that, iron can be stored in the liver joined to ferritin, which is an iron storage protein. The mechanisms regulating systemic iron homeostasis are largely centered on the liver and involve two molecules, hepcidin and ferroportin, that work together to regulate the flow of iron from cells into the systemic circulation [2]. 

Due to the key role of iron in many physiological cell functions, including replication, ATP, DNA synthesis and the heme group in hemoglobin [3], and as a constituent of essential cofactors such as iron–sulfur (Fe-S) clusters [4], the organism has evolved to conserve body iron stores; however, it does not have an efficient mechanism for removing excess iron, iron-overload being highly deleterious to many physiological mechanisms. In addition, defects in molecules related to regulating iron homeostasis are usually a cause of genetically inherited iron overload, called hereditary hemochromatosis (HH) [2]. Therefore, it is essential to tightly regulate iron homeostasis, a function performed mainly by the liver [5].

The liver is one of the most functionally and metabolically active organs in the body. In addition to roles in detoxification, digestion, protein synthesis, gluconeogenesis, and fat metabolism, the liver also plays a significant role in iron homeostasis. It is responsible for approximately 8% of plasma iron turnover and it is the major site for iron storage, acting also as the key regulator of iron homeostasis [6]. The liver synthesizes hepcidin, a peptide which binds to and induces the internalization of ferroportin [7], reducing the amount of iron released into the bloodstream, therefore being the major contributor to systemic regulation of iron status [8]. The noteworthy influence of the liver on body iron regulation can be attributed to the expression of many liver-specific or liver-enriched proteins, which play an important role in the physiological regulation of iron metabolism [6].

On the other hand, it has been previously reported that fermented goat milk consumption improves the key proteins of intestinal iron metabolism during IDA recovery, enhancing the digestive and metabolic utilization of iron [9]. However, in spite of the crucial role of the liver as a central regulator of iron metabolism, to date, no studies have directly tested the modulation of hepatic gene and protein expression profiles during anemia recovery, including divalent metal transporter 1 (DMT1), ferritin light chain 1 (FTL1), ferroportin 1 (FPN1), and hepcidin antimicrobial peptide (HAMP). Taking into account all these considerations, the aim of this study was to contribute to a better understanding of iron metabolism during IDA recovery, by studying how fermented goat vs. cow milk (the most commonly consumed milk worldwide) consumption affects liver iron homeostasis during nutritional iron repletion in animal models with severe, induced iron-deficiency anemia and overload.

## 2. Material and Methods 

### 2.1. Animals and Experimental Design

One hundred male Wistar rats aged 3 weeks and weighing about 34.56 ± 6.35 g, purchased from the University of Granada Laboratory Animal Service (Granada, Spain), were included in this study. The animals were maintained under standard animal housing conditions with a 12-hour light/dark cycle (lights on at 9:00 A.M.), temperature (23 ± 2 °C) and humidity (60 ± 5%). All animal experiments were carried out in accordance with Directive 2010/63/EU on the protection of animals used for scientific purposes.

**Figure 1:**
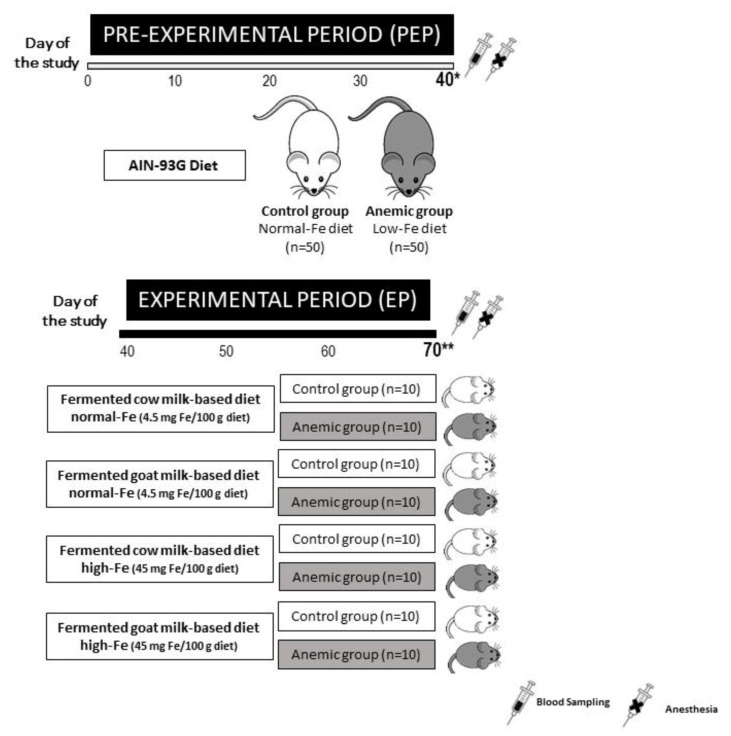
Experimental design of the study. * 10 animals per group and ** all the animals were anesthetized, peripheral blood samples from caudal vein were analyzed for hematological and biochemical parameters and the liver was removed.

In the pre-experimental period (PEP), 100 rats were randomly divided into two groups; a control group received the AIN-93G diet (*n* = 50) [10] and an anemic or experimental group received a low Fe diet (*n* = 50) for 40 days [11]; deionized water and diet were available ad libitum. In the experimental period (EP), control and anemic groups were fed for 30 days with either fermented cow milk or fermented goat milk diet, with normal Fe (4.5 mg/100 g) or high Fe content (45.0 mg/100 g) (Fe citrate) [12]. Deionized water was available ad libitum, and dietary intake pattern induced by pair feeding (80% of the average intake) (Figure 1). At the end of the PEP and EP, hematological parameters and serum iron, total iron binding capacity (TIBC), transferrin saturation, ferritin, serum hepcidin and transaminases were determined. Furthermore, the liver was removed (at the end of the PEP, in 10 animals per group and in all the animals in the EP), for measurement of hepatosomatic index (HSI), and a fraction of the liver was snap-frozen in liquid nitrogen and keep in a −80 °C freezer for subsequent mineral analysis (liver iron content). Subsequently 1 g of liver was stored overnight at 4 °C with RNA-later (Thermo Fisher Scientific, Waltham, MA, USA), the solution was removed and stored at −80 °C until isolation of total RNA. 

### 2.2. Diets Preparation

Diets were prepared with fermented cow or fermented goat milk. *Lactobacillus bulgaricus* subsp. *delbrueckii* and *Streptococcus thermophilus* were inoculated to an initial concentration of 1 × 10^11^ CFU/mL (10 mL/L inoculum) in goat or cow milk, and then the samples were incubated at 37 °C for approximately 24 h. Subsequently, fermented milk samples were dehydrated by a smooth industrial process to obtain products with a moisture content ranging between 2.5% and 4.5%. Sufficient amounts of fermented dehydrated cow or goat milk were utilized in the experimental diets to provide 20% of protein and 10% of fat. The constituents and nutrient compositions of the experimental diets are presented in Table 1.

### 2.3. Hematological Tests

Hematological parameters were measured at the end of PEP and EP using a fully automated hematology analyzer (Mythic 22CT, C2 Diagnostics, Grabels, France).

### 2.4. Transferrin Saturation, Serum Iron, Total Iron Binding Capacity (TIBC), Serum Ferritin and Serum Hepcidin 

Transferrin saturation, serum iron and TIBC were determined using Sigma Diagnostics Iron and TIBC reagents (Sigma-Aldrich Co., St. Louis, MO). Serum ferritin was measured by the Elisa method using a standard kit (rat ferritin ELISA Kit) supplied by Biovendor GmbH, Heidelberg (Germany). Hepcidin-25 was determined using a DRG ELISA Kit (DRG Instruments GmbH, Germany). 

### 2.5. Hepatosomatic Index, Hepatic Iron Concentration and Transaminases Analysis

The HSI was determined by the use of the following equation:

HSI = (weight of the liver/weight of the body) × 100

Prior to iron analysis, liver fractions were mineralized by wet digestion in a sand bath (Selecta, Barcelona, Spain) using nitric acid followed by a mixture of HNO3:HClO4, 1:4 *v*/*v* (69%:70%, *v*/*v*; Merck, Darmstadt, Germany) until the total elimination of organic matter. Finally, the samples were diluted with Milli Q (Millipore S.A., Bedford, MA, USA) ultrapure water. Iron analysis was undertaken using a PerkinElmer Optima 8300 inductively coupled plasma-optical emission spectrometer (ICP-OES) (Waltham, MA, USA) with a segmented-array charge-coupled device (SCD) detector. Multi-elemental Atasol calibration solution (Analytika, Khodlova, Prague) was used to calibrate the apparatus. For the calibration curve, diluted standards were prepared from concentrated standard solutions. After each series of 5 samples, an internal standard solution of 10 mg/L was used. The acceptable result was assessed as 10%. Three replicates of each sample were analyzed.

Alanine aminotransferase (ALT) and aspartate aminotransferase (AST) were measured by standard colorimetric and enzymatic methods using a BS-200 Chemistry Analyzer (Shenzhen Mindray Bio-Medical Electronics Co. Ltd., Shenzhen, China). 

### 2.6. RNA Extraction and Quantitative Real Time PCR

From liver samples, total RNA was extracted with TRIsure lysis reagent (Bioline, Luckenwalde, Germany) following the manufacturer’s instructions. The RNA quantity and purity were measured using a spectrophotometer (NanoDrop 1000, Thermo Fisher Scientific, Waltham, Massachusetts, USA) at 260/280 nm. Reverse transcription was performed on 1 µg of total RNA in a 20 µL reaction to synthesize complementary DNA (cDNA), using an iScript cDNA Synthesis kit (Bio-Rad). Quantitative real-time PCR was performed in a total reaction volume of 20 µL using the CFX96 Touch Real-Time PCR Detection System (Bio-Rad) and SYBR Green detection using Sso Avdvanced Universal SYBR Green Supermix (Bio-Rad). Primer sequences of divalent metal transporter 1 (DMT1), ferroportin 1 (FPN1), ferritin (FTL1) and hepcidin antimicrobial peptide (HAMP) for quantitative real-time PCR are shown in Table 2 and were obtained from Eurofins MWG Biotech (Ebersberg, Germany). The expression of the target genes was normalized to the housekeeping gene β-actin. All the measurements were done in duplicate and, to confirm PCR product size, melt curve analysis and gel electrophoresis were conducted.

### 2.7. Western Blotting and Immunocytochemistry

Liver samples were mechanically homogenized in tissue protein extraction reagent (T-PER) (Thermo Scientific Inc., Hanover Park, IL, USA) supplemented with a protease inhibitor cocktail (Sigma-Aldrich, St. Louis, MO, USA) under ice-cold conditions. On 4%–20% Criterion TGX (Tris-Glycine extended) gels (Mini-PROTEAN TGX Precast Gels; Bio-Rad), 12 µg of total protein was separated in a vertical electrophoresis tank (Mini-PROTEAN System; Bio-Rad) at 250 V for 20 min. Separated proteins were transferred onto a polyvinylidene difluoride membrane (Bio-Rad) by wet transfer for 60 min at 120 V. Thereafter, the membranes were blocked with 5% dry milk in Tris-buffered saline (TBS) plus Tween-20 (TTBS) (Bio-Rad) solution for 1 hour at room temperature. After three washes in TBS, membranes were incubated with rabbit anti-DMT1 polyclonal, dilution 1:400 (Santa Cruz Biotechnology Inc., Santa Cruz, CA, USA), rabbit anti-SLC40A1 polyclonal antibody (FPN1), dilution 1:800, hepcidin polyclonal antibody, dilution 1:500, and mouse anti-β-actin monoclonal, dilution 1:1000 (Abcam, UK) as primary antibodies, in 5% dry milk in TTBS overnight at 4 °C with shaking. β-actin was used as the loading control. Then, the membranes were washed 3 times in TTBS and incubated for 1 h at room temperature with the appropriate secondary conjugated antibody Immun-Star Goat Anti-Rabbit (GAR)-HRP; Bio-Rad Laboratories; 1:40,000 amd ImmunStar Goat Anti-Mouse (GAM)-HRP; 1:80,000 in TTBS. Immunoblots were detected with a chemiluminescence Luminata forte western HRP Substrate (Merck KGaA, Darmstadt, Germany) and visualized with chemiluminescence using ImageQuant LAS 4000 (Fujifilm Life Science Corporation, USA). All results were analyzed with Image J software.

### 2.8. Statistical Analysis

Data are presented as mean ± standard error of the mean (SEM) and statistical analyses were performed using SPSS 26.0 (SPSS Inc., Chicago, IL, USA). Differences between groups (control versus anemic during the PEP and normal Fe versus high Fe during the EP) were tested for statistical significance with Student’s *t*-test. Following a significant F-test (*p* <0.05), individual means were tested by pairwise comparison with Tukey’s multiple comparison test when main effects and interactions were significant. Two-way analysis of variance (ANOVA) was used to determine the effect of the type of diet supplied to the animals, anemia, and iron content in the diet. Statistical significance was set at *p* < 0.05 for all comparisons.

## 3. Results

### 3.1. Effect of Iron Deficiency on Hepatosomatic Index, Liver Iron Content and Serum Levels of Aspartate Aminotransferase and Alanine Aminotransferase

Iron deprivation impaired all the hematological parameters studied [9], and these parameters are detailed in Supplementary Table S1. Additionally, body weight and liver iron content were lower in the anemic group (*p* < 0.001); however, liver weight remained unchanged, and, as a consequence, HSI was higher in anemic group (*p* < 0.05). Transaminases were significantly higher in the anemic group (*p* < 0.001) (Table 3).

### 3.2. Effect of Fermented Milk-Based Diets on Hepatosomatic Index, Liver Iron Content and Serum Levels of Aspartate Aminotransferase and Alanine Aminotransferase during Anemia Recovery

After supplying both fermented milk-based diets during a month, all the hematological parameters were recovered, either with normal Fe or high Fe content. The results were described [9] and are shown in Supplementary Table S2. Fermented goat milk reduced body weight in control and anemic animals, either with normal Fe or high Fe content (*p* < 0.001) compared to animals fed fermented-cow-milk-based diets. Body weight was significantly lower in anemic animals fed both fermented milks with normal Fe content (*p* < 0.01). Body weight was lower a with high Fe content diet than with a normal Fe content diet in animals fed with fermented-cow-milk-based diets in both groups (control and anemic) (*p* < 0.05). HSI was higher in both groups of animals (control and anemic) fed fermented-goat-milk-based diets, either with normal Fe or high Fe, compared to animals fed fermented-cow-milk-based diets (*p* < 0.001). Liver iron content was increased in animals fed with fermented goat milk compared to animals fed with fermented cow milk with normal Fe content diets (*p* < 0.01). In contrast, liver iron content was lower in animals fed fermented goat milk compared to fermented cows’ milk with high Fe content (*p* < 0.01). Liver iron content was lower in anemic animals compared to the control group, irrespective of the iron content in both types of fermented milks (*p* < 0.01). As expected, dietary high Fe content increased the liver content of this mineral in control and anemic animals fed fermented cow milk (*p* < 0.001), this increase being lower in the animals fed fermented goat milk (*p* < 0.05). AST and ALT were lower in rats fed with fermented-goat-milk-based diets compared to rats fed with fermented-cow-milk-based diets in all experimental conditions (*p* < 0.01). High Fe content did not affect the transaminases when supplying both milk-based diets (Table 4).

### 3.3. Effect of Fermented Milk-Based Diets on Liver Iron Homeostasis during Anemia Recovery

**Figure 2:**
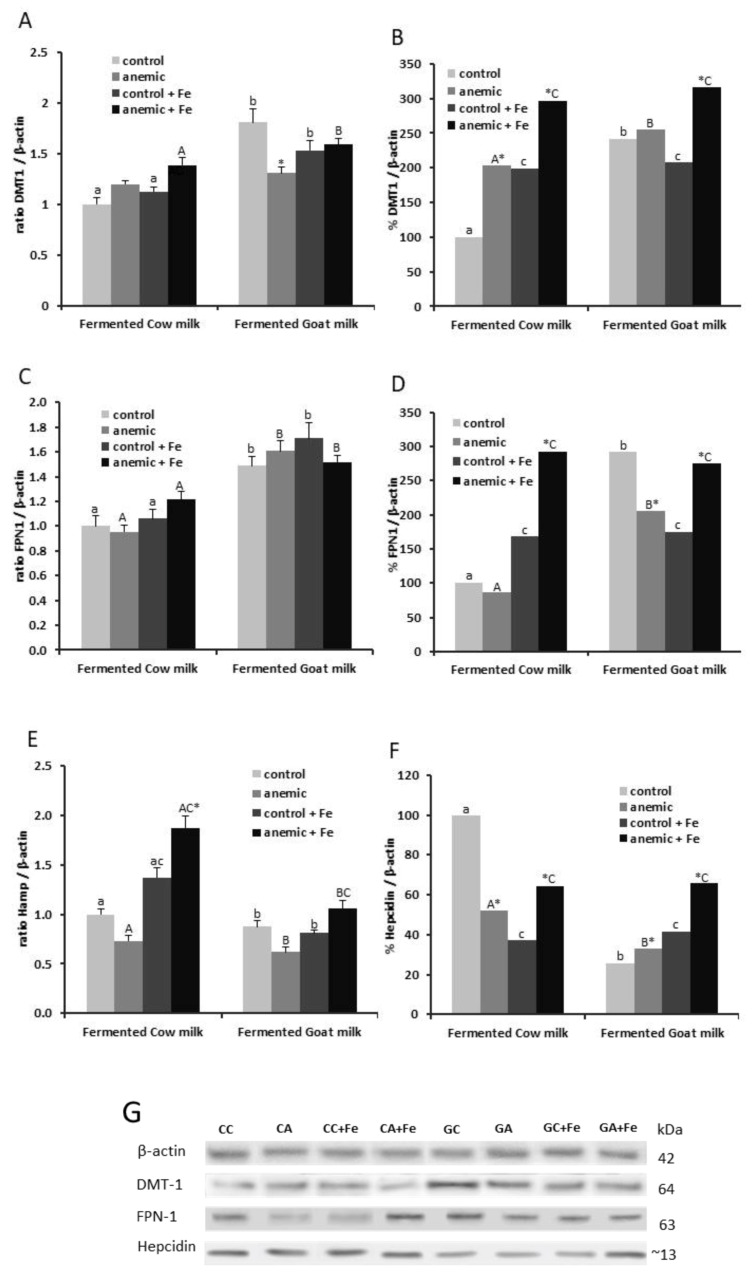
mRNA levels (**A**,**C**,**E**) and protein expression levels (**B**,**D**,**F**) of DMT-1, FPN1 and hepcidin in livers of control and anemic rats, fed normal Fe or high Fe content fermented cow or goat milk-based diets. Values are represented as mean ± SEM (*n* = 10). For protein expression, values are expressed as % vs β-actin. a,b: mean values among groups of control rats fed with different diets; different lower-case letters in the same row indicate a significant difference by two-way ANOVA (Tukey’s test). A,B: Mean values among groups of anemic rats fed with different diet; different upper-case letters in the same row indicate a significant difference by two-way ANOVA (Tukey’s test). * Significantly different (*p* < 0.05) from the control group by Student’s *t*-test. c: Mean values of control rats were significantly different from the corresponding groups of rats fed with normal Fe content at *p* < 0.05 by Student’s *t*-test. C: Mean values of anemic rats were significantly different from the corresponding group of rats fed with normal Fe content at *p* < 0.05 by Student’s *t*-test. (**G**) Representative immunoblots. Abbreviations: cow control (CC), cow anemic (CA), cow control high Fe content (CC+Fe), cow anemic high Fe content (CA+Fe), goat control (GC), goat anemic (GA), goat control high Fe content (GC+Fe), goat anemic high Fe content (GA+Fe).

Fermented goat milk up-regulated liver DMT1 gene expression in control and anemic rate fed with high Fe content (*p* < 0.01) and in control groups fed with a normal Fe diet, and previously induced anemia reduced DMT1 expression in the animals fed fermented goat milk with normal Fe content (*p* < 0.05) (Figure 2A). Similarly, DMT1-relative protein expression was higher in control and anemic animals fed fermented goat milk with normal Fe (*p* < 0.001). Induced anemia increased liver DMT1 protein expression in animals fed both fermented milk with normal Fe content (*p* < 0.001). High Fe content increased DMT1 protein expression in all groups of animals fed fermented goat or cow milk (*p* < 0.001), except in control animals fed fermented goat milk, which showed a decrease (*p* < 0.05) (Figure 2B).

Expression of FPN1 mRNA increased in control and anemic animals fed fermented goat either with normal Fe (*p* < 0.001) or high Fe content (*p* < 0.01) (Figure 2C). Protein expression of FPN1 increased in control and anemic animals fed fermented goat milk with normal Fe content (*p* < 0.001). Anemia decreased FPN1 protein expression in animals fed fermented goat milk with normal Fe (*p* < 0.01) and increased in animals fed both fermented milks with high Fe content (*p* < 0.001). High Fe content increased liver FPN1 protein expression in all groups of animals fed fermented goat or cow milk (*p* < 0.001), except control animals fed fermented goat milk, which showed a decrease (*p* < 0.001) (Figure 2D).

HAMP mRNA expression was lower in control and anemic animals fed fermented goat milk with normal Fe (*p* < 0.05) and also in control and anemic animals fed fermented goat milk with high Fe content (*p* < 0.001). Anemia increased HAMP mRNa expression in animals fed fermented cow milk with high Fe content (*p* < 0.01). High Fe content increased HAMP gene expression in anemic animals fed both fermented milks (*p* < 0.001) and in control groups fed a fermented cow milk diet (*p* < 0.01) (Figure 2E). Hepcidin protein expression decreased in control and anemic animals fed fermented goat milk with normal Fe content (*p* < 0.001). Anemia decreased hepcidin protein expression in animals fed fermented cow milk with normal Fe content (*p* < 0.001) and increased in animals fed fermented goat milk with normal Fe content (*p* < 0.05); however, with high Fe content, anemia increased hepcidin protein expression in the animals fed both fermented milks (*p* < 0.001). High Fe content increased hepcidin protein expression in all groups of animals fed fermented cow or goat milk (*p* < 0.01) but decreased in control animals fed fermented cow milk (*p* < 0.001) (Figure 2F).

**Figure 3:**
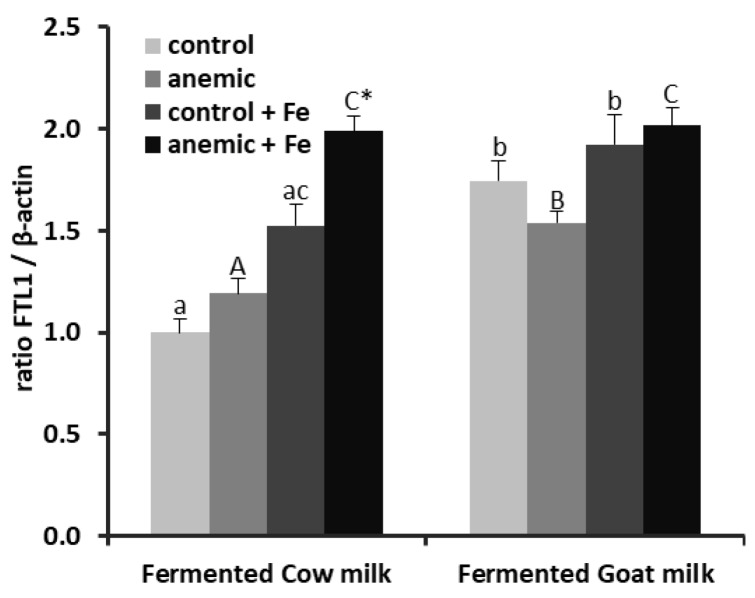
mRNA levels of FTL1 in the liver of control and anemic rats, fed normal Fe or high Fe content fermented cow- or goat-milk-based diets. Values are represented as mean ± SEM (*n* = 10). a,b: mean values among groups of controls rats fed with different diets; different lower case letters in the same row indicate a significant difference by two-way ANOVA (Tukey’s test). A,B: mean values among groups of anemic rats fed with different diets; different upper case letters in the same row indicate a significant difference by two-way ANOVA (Tukey’s test). * Significantly different (*p* < 0.05) from the control group by Student’s *t*-test. c: mean values of controls rats were significantly different from the corresponding group of rats fed with normal Fe content at *p* < 0.05 by Student’s *t*-test. C: mean values of anemic rats were significantly different from the corresponding group of rats fed with normal Fe content at *p* < 0.05 by Student’s *t*-test.

In general, fermented goat milk induced an up-regulation of mRNA FTL1 expression in control and anemic animals fed either with normal Fe or high Fe content (*p* < 0.01). Anemia up-regulated liver mRNA FTL1 expression in animals fed fermented cow milk with high Fe content (*p* < 0.01). High Fe content up-regulated FTL1 mRNA expression in the anemic groups fed both fermented milks (*p* < 0.001) and in the control group fed fermented cow milk (*p* < 0.01) (Figure 3). 

## 4. Discussion

Anemia reduced body weight in the PEP, due to the impairment of hematological parameters and the depletion of the hepatic iron levels. These negatively affect the weight gain of animals during the growing period, since the hypoxia induced by the lack of iron limits ATP production and decreases levels of thyroid hormones and ghrelin, inducing cachexia and leading to reductions of lean mass [13]. In a previous study, we reported that serum total bilirubin was increased in hepatocellular damage. In that study, bilirubin was increased and AST and ALT activities were also found elevated in IDA. The increase of transaminase release into the bloodstream could be due to the impairment of the hepatic function induced by the iron deficiency [13,14].

During the EP, body weight was statistically lower in the animals fed fermented goat milk. This finding can be explained because, as previously reported [15], fermented goat milk consumption influences adipose tissue depot homeostasis during iron deficiency recovery, reducing adiposity, increasing leptin elevation and reducing ghrelin, and, therefore, diminishing appetite and increasing basal metabolic rate. Additionally, fermented goat milk consumption reduces adiponectin levels and shows an inverse correlation with non-esterified fatty acids, indicating increased lipolysis rates in adipose tissue [15]. 

Liver iron homeostasis is tightly regulated to maximize the iron supply when anemia is established and to promote storage when iron status is adequate. Intracellular iron content is regulated by the relative rate of cellular import, via DMT1, versus export, via FPN1 [16].

Fermented goat milk improves iron metabolism, because, as previously reported [15], it increases expression of duodenal key proteins in intestinal iron metabolism, improving iron absorption after induced anemia. However, in spite of the role of the liver as the central regulator of iron homeostasis, a better characterization of the hepatic events in the pathophysiology of iron deficiency instauration and recovery leads to new nutritional strategies to improve liver molecular functions related to iron homeostasis.

After supplying the fermented-milk-based diets, iron repletion was more efficient with fermented goat milk, a fact that is corroborated by the recovery of hematological parameters and the liver iron content recorded in the current study. This fact can be explained due to the beneficial increase in the liver DMT1 iron-transport gene and protein expression, enhancing iron metabolism and storage compared with fermented cow milk. DMT1 is localized both to the plasma membrane and in the cytosol of hepatocytes [17]. In addition to its role in endosomal iron export, the presence of DMT1 on the microvillous membrane of hepatocytes reveals that the liver is capable of absorbing non-heme, non-transferrin-bound iron. Contrary to the duodenal DMT1, hepatic DMT1 expression parallels iron status [17], playing a key role by regulating iron homeostasis in the liver. Therefore, the increased liver DMT1 protein levels faithfully reflect the increased liver iron storage after dietary iron repletion [18] and, this way, the increase in DMT1 protein expression in anemic animals fed fermented goat milk reveals that iron repletion is more efficient, a finding that is supported and corroborated by the higher iron storage in the rats fed fermented goat milk compared with fermented cow milk. As previously reported, goat milk fat is richer in medium-chain triglycerides, which are oxidized in the mitochondria, providing fast energy discharge used in several metabolic pathways [19] and thus contributing to increasing the synthesis of carrier proteins such as DMT1 [20]. On the other hand, goat milk has more than twice the vitamin A content of cow milk [19], and this vitamin increases liver DMT1 protein expression by posttranscriptional regulation via increased protein translation or decreased degradation [18].

Hepcidin is a key indicator of iron status and metabolism. This hormone regulates iron levels and location in response to nutritional status. High hepcidin levels block intestinal iron absorption and macrophage recycling, causing anemia. Low hepcidin levels favor bone marrow iron supply for hemoglobin synthesis and erythropohiesis. Multiple factors regulate the expression of hepcidin in the liver. As expected, low serum hepcidin levels were recorded in the PEP due to the increased expression of key duodenal proteins involved in iron absorption [20], favoring red blood cell production during insufficient dietary iron supply. On the other hand, inflammation is a strong inducer of hepcidin production and release from the liver [21,22,23], resulting in reduced iron release from stores and macrophages, thereby reducing iron in the circulation and disrupting iron homeostasis [24]. In the current study, a down-regulation of liver hepcidin has been recorded in animals consuming fermented goat milk, a fact that increases the iron efflux from the hepatocytes to the serum, due to the inverse correlation between hepcidin expression and the FPN1 activity [9,25]. 

In addition, we have previously reported that fermented goat milk consumption decreased pro-inflammatory cytokines and increased anti-inflammatory cytokines [25], due to the anti-inflammatory activities of its biologically active lipid fractions, such as sphingomyelin, phosphatidylcholine, and phosphatidylethanolamine lipid derivatives [26,27]. Moreover, fermented goat milk has a higher content in conjugated linoleic acid than fermented cow milk, featuring a putative role modulating anti-inflammatory responses [25]. All these anti-inflammatory properties of fermented goat milk would contribute to the decrease in liver hepcidin expression. This decrease in hepcidin, together with the increase in DMT1 and FPN1 gene and protein liver expression recorded when fermented goat milk is supplied, would led to the mobilization of iron from hepatic storage to sustain erythropoiesis and improve iron status, even under Fe-overload conditions. These data indicate a not only a better hematological recovery but also improved iron mobilization for liver storage to target organs.

Although, in physiological conditions, hepatocytes have a high capacity for iron storage, the liver is also the main organ affected by the oxidative stress caused by Fe-overload toxicity due to its high propensity to induce reactive oxygen species (ROS) [28]. As mentioned above, the increase in DMT1 and FPN1 gene and protein liver expression recorded with fermented goat milk lead to lower amounts of iron stored in the hepatocytes during the dietary Fe overload in comparison with fermented cow milk, reducing the evoked oxidative stress. In this sense, fermented goat milk has positive effects on enzymatic antioxidant hepatic defense, even in a situation of Fe overload, which limits the processes of lipid peroxidation in comparison with cow milk, due to the improvement of zinc bioavailability (with antioxidant capacity) and the better lipid quality in comparison to fermented cow milk, reducing the generation of ROS [29]. Additionally, these findings are supported by the previous results [30], reporting a clear diminishment of transaminase release into the bloodstream, corroborating the hepatoprotective effect of goat milk during Fe overload and reducing hepatocellular damage, a fact that could avoid, at least partially, the progression of Fe-overload-related diseases [31].

FTL1 is the intracellular iron storage protein that stores and releases iron in a controlled way, and its expression greatly increases when cellular iron concentrations rise, providing the cell with an enormous ability to sequester iron. When intracellular iron content is low, iron-responsive element-binding protein 2 (IRP2) represses FTL1, mRNA translation, while intracellular iron accumulation promotes IRP2 degradation and allows FTL1 mRNA translation [32]. As previously mentioned, the increased DMT1 and FPN1 expression levels when fermented goat milk is supplied improve iron storage, also explaining the higher levels of mRNA FTL1. In addition, Fe overload increased FTL1 gene expression, probably as a compensatory mechanism to avoid the liver oxidative damage induced by Fe overload, because intracellular iron redox activity is controlled by ferritin [33].

## 5. Conclusions

Fermented goat milk consumption potentiates the up-regulation of key genes and proteins involved in iron metabolism, such as DMT1 and FPN1, and downregulates liver hepcidin, enhancing and improving iron repletion during anemia recovery. In addition, taking into account the better iron storage in the liver during anemia recovery, the reduction in the iron accumulation during Fe overload, and the improvement of the hematological parameters, fermented goat milk consumption induces a hepatic physiological adaptation of the key genes and proteins that regulate the fluctuation of the cellular iron levels, favoring whole-body iron homeostasis.

## Figures and Tables

**Table 1:** Composition of experimental diets.

Constituents	Pre-Experimental Period	Experimental Period	
g/100 g Diet		Fermented Milk Diets ^2^	
	AIN-93G ^1^	Cow Milk	Goat Milk
Protein	20.00	20.50	20.60
Lactose	-	29.50	29.10
Fat	10.00	10.00	29.10
Wheat starch	50.00	20.00	20.30
Constant ingredients ^3^	20.00	20.00	20.00

^1^ Normal iron content for control rats (4.5 mg Fe/100 g diet) [10], or low iron content (0.5 mg Fe/100 g diet) [11] for the anemic group. ^2^ Specific vitamin and mineral premix supplements for fermented goat and cow milk diets were to meet the recommendations of the AIN-93G for diets with normal iron (4.5 mg Fe/100 g diet) [12] or diets with high iron content (45 mg Fe/100 g diet) (Raja et al., 1994). ^3^ Fibre (micronized cellulose) 5%, sucrose 10%, choline chloride 0.25%, L-cystine 0.25%, mineral premix 3.5%, vitamin premix 1%.

**Table 2:** Primers, annealing temperatures, and product sizes for PCR amplification.

Gene	Direction	Primer Sequence (5′→3′)	Annealing Temperature	Size (bp)
β-Actin	Forward	GGGGTGTTGAAGGTCTCAAA	57 °C	165
	Reverse	TGTCACCAACTGGGACGATA		
DMT1	Forward	GGCATGTGGCACTGTATGTG	59 °C	163
	Reverse	CCGCTGGTATCTTCGCTCAG		
FPN1	Forward	GAACAAGAACCCACCTGTGC	57 °C	191
	Reverse	AGGATGGAACCACTCAGTCC		
HAMP	Forward	CCTATCTCCGGCAACAGACG	59 °C	121
	Reverse	GGGAAGTTGGTGTCTCGCTT		
FTL1	Forward	GCCCTGGAGAAGAACCTGAA	59 °C	247
	Reverse	AGTCGTGCTTCAGAGTGAGG		

**Table 3:** Hepatosomatic index, liver iron content and serum levels of aspartate aminotransferase and alanine aminotransferase from control and anemic rats in the pre-experimental period (PEP).

	Control Group (*n* = 20)	Anemic Group (*n* = 20)
Body weight (g)	239.7 ± 3.9	201.15 ± 2.9 **
Liver weight (g)	6.324 ± 0.31	6.129 ± 0.31
HSI (%)	2.55 ± 0.07	2.89 ± 0.09 *
Liver iron content (µg/g dry weight)	615.25 ± 31.10	432.31 ± 24.07 **
AST (UI/L)	103.58 ± 8.93	228.04 ± 18.45 **
ALT (UI/L)	24.57 ± 1.16	52.28 ± 2.73 **

Values are means ± SEM (*n* = 10). HSI, hepatosomatic index; AST, aspartate aminotransferase; ALT, alanine aminotransferase. * Significantly different from the control group (*, *p* < 0.05) Student’s *t*-test). ** Significantly different from the control group (**, *p* < 0.001) Student’s *t*-test).

**Table 4:** Hepatosomatic index, liver iron content and serum levels of aspartate aminotransferase and alanine aminotransferase, from control and anemic rats fed for 30 days with fermented cow- or goat-milk-based diets with normal Fe or high Fe content in the experimental period (EP).

		Fermented Cow Milk		Fermented Goat Milk		2-WAY ANOVA		
	Fe Content	Control Group	Anemic Group	Control Group	Anemic Group	Diet	Anemia	Fe Content
Body weight (g)	Normal	365.23 ± 8.61 ^a^	347.21 ± 8.39 ^A,^*	278.98 ± 3.70 ^b^	255.41 ± 2.85 ^B,^*	<0.001	<0.01	<0.05
	High	339.42 ± 5.18 ^a,c^	329.22 ± 5.81 ^A,C^	287.27 ± 4.92 ^b^	267.57 ± 4.03 ^B^	<0.001	NS ^1^	
Liver weight (g)	Normal	6.528 ± 0.24 ^a^	6.269 ± 0.10 ^A^	8.391 ± 0.23 ^b^	8.521 ± 0.21 ^B^	<0.001	NS	<0.05
	High	6.764 ± 0.2 ^a^	6.555 ± 0.12 ^A^	7.692 ± 0.22 ^b,c^	7.934 ± 0.22 ^B,C^	<0.01	NS	
HSI (%)	Normal	1.84 ± 0.04 ^a^	1.77 ± 0.02 ^A^	2.95 ± 0.03 ^b^	3.27 ± 0.05 ^B^	<0.001	NS	NS
	High	1.79 ± 0.03 ^a^	1.82 ± 0.03 ^A^	2.65 ± 0.04 ^b^	3.12 ± 0.04 ^B^	<0.001	NS	
Liver iron content (µg/g dry weight)	Normal	559.56 ± 28.72 ^a^	401.56 ± 24.50 ^A,^*	666.45 ± 33.21 ^b^	489.32 ± 29.64 ^B,^*	<0.01	<0.01	<0.01
	High	832.25 ± 32.56 ^a,c^	782.32 ± 33.55 ^A,C,^*	735.67 ± 29.33 ^b,c^	657.15 ± 29.22 ^B,C,^*	<0.01	<0.01	
AST (UI/L)	Normal	107.62 ± 4.29 ^a^	80.86 ± 4.25 ^A,^*	67.99 ± 2.75 ^b^	61.11 ± 2.12 ^B^	<0.01	<0.05	NS
	High	82.92 ± 4.15 ^a,c^	78.19 ± 3.82 ^A^	60.43 ± 1.10 ^b^	68.47 ± 2.03 ^B^	<0.01	NS	
ALT (UI/L)	Normal	28.91 ± 1.34 ^a^	27.77 ± 3.91 ^A^	23.14 ± 1.9 ^b^	16.49 ± 0.76 ^B,^*	<0.01	<0.01	NS
	High	24.0.4 ± 1.73 ^a^	19.00 ± 1.21 ^A^	19.47 ± 0.53 ^b^	14.48 ± 0.35 ^B,^*	<0.01	<0.01	

Values are means ± SEM (*n* = 10). ^1^ NS, not significant. HSI, hepatosomatic index; AST, aspartate aminotransferase; ALT, alanine aminotransferase. * Significantly different from the control group (*p* < 0.05) *Student’s t*-test). ^a,b^ Mean values among groups of controls rats fed with different diets; different lower-case letters in the same row indicate a significant difference by two-way ANOVA (Tukey’s test). ^A,B^ Mean values among groups of anemic rats fed with different diets; different upper-case letters in the same row indicate a significant difference by two-way ANOVA (Tukey’s test). ^c^ Mean values of controls rats were significantly different from the corresponding group of rats fed with normal Fe content at *p* < 0.05 by Student’s *t*-test. ^C^ Mean values of anemic rats were significantly different from the corresponding group of rats fed with normal Fe content at *p* < 0.05 by Student’s *t*-test.

## Supplementary Materials

The following are available online at https://www.mdpi.com/2072-6643/12/5/1336/s1, Table S1: Hematological parameters of control and anemic rats in the pre-experimental period (PEP); Table S2. Hematological parameters from control and anemic rats fed for 30 days with fermented cow or goat milk-based diets with normal-Fe content or Fe-overload in the experimental period (EP).
