# Supplementary material for: Role of Fermented Goat Milk on Liver Gene and Protein Profiles Related to Iron Metabolism during Anemia Recovery
Source: Nutrients. 2020 May 8;12(5):1336. doi: 10.3390/nu12051336 (PMC7284947; doi:10.3390/nu12051336)
Supplement: Supplementary file 1 [file nutrients-12-01336-s001.pdf]

Supplementary Material

Table S1. Hematological parameters of control and anemic rats in the pre-experimental period (PEP).

Hematological parameters	Control group (n = 40)	Anemic group (n = 40)
Total blood		
Hb concentration (g/L)	133.88 ± 2.98	60.22 ± 2.87 *
RBCs (10^{12}/L)	7.14 ± 0.20	3.08 ± 0.24 *
Hematocrit (%)	40.01 ± 1.13	12.76 ± 1.33 *
MCV (fL)	55.76 ± 0.53	36.91 ± 0.37 *
MCH (pg)	19.47 ± 0.15	14.10 ± 0.63 *
MCHC (g/dl)	35.54 ± 0.36	30.21 ± 0.82 *
RDW (%)	16.23 ± 0.35	19.16 ± 0.39 *
Platelets (10^9/L)	733 ± 72.15	2123 ± 119 *
WBCs (10^9/L)	8.91 ± 0.39	8.53 ± 0.88
Lymphocytes (10^6/ml)	7.98 ± 0.58	5.76 ± 0.84 *
Serum		
Fe (µg/L)	1336 ± 99.12	601 ± 55.98 *
TIBC (µg/L)	2675 ± 189	17935 ± 598 *
Transferrin saturation (%)	49.02 ± 5.87	3.93 ± 0.41 *
Ferritin (µg/L)	79.74 ± 2.12	49.65 ± 1.64 *
Hepcidin, ng/mL	16.87 ± 0.46	13.48 ± 0.62 *

Values are means ± SEM. Hb, hemoglobin; RBCs, red blood cells; MCV, mean corpuscular volume; MCH, mean corpuscular Hb; MCHC, mean corpuscular Hb concentration; RDW, red cell distribution width; WBCs, white blood cells; TIBC, total Fe-binding capacity. *Significantly different from the control group (*, $p < 0.001$) Student's t test).

Table S2. Hematological parameters from control and anemic rats fed for 30 days with fermented cow or goat milk-based diets with normal-Fe content or Fe-overload in the experimental period (EP).

Hematological parameters		Fermented cow milk		Fermented goat milk		2-WAY ANOVA		
	Fe content	Control group	Anemic group	Control group	Anemic group	Diet	Anemia	Fe content
Hb concentration (g/l)	Normal	129.55 ± 2.89	129.33 ± 2.61	131.95 ± 2.76	129.15 ± 2.48	NS[1]	NS	< 0.001
	Overload	142.63 ± 2.63c	141.10± 2.92AC	141.30 ± 2.98c	147.35 ± 3.01BC	< 0.05	NS	
RBCs (10^{12}/l)	Normal	7.06± 0.18	7.08± 0.22	7.39 ± 0.21	7.21± 0.20	< 0.05	NS	< 0.05
	Overload	6.94 ± 0.17a	7.19 ± 0.24	8.01 ± 0.30 bc	7.11 ± 0.21	< 0.01	NS	
Haematocrit (%)	Normal	40.02± 1.19a	39.01± 0.97A	41.93± 1.23b	42.95 ± 0.98B	< 0.01	NS	< 0.01
	Overload	39.37 ± 1.33a	44.89 ± 2.76C	44.86 ± 1.26 bc	45.42 ± 1.35C	< 0.05	NS	
MCV (fL)	Normal	57.68 ± 0.54	55.34 ± 0.59	57.25 ± 0.56	55.04 ± 0.53	NS	NS	NS
	Overload	56.79 ± 0.58	53.18± 0.55A	56.44 ± 0.53	56.15± 0.53B	< 0.05	NS	
Platelets (10^9/l)	Normal	933.00 ± 70.32	963.00 ± 66.45	926.00 ± 79.65	935.33 ± 66.89	NS	NS	NS
	Overload	939.67 ± 71.37	965.50 ± 72.22	933.59 ± 81.32	945.86 ± 70.26	NS	NS	
Serum Fe (µg/l)	Normal	1346 ± 86.18	1355 ± 86.35	1352 ± 88.96	1326 ± 94.35	NS	NS	< 0.01
	Overload	1591± 100c	1587 ± 102C	1556 ± 99c	1576 ± 97C	NS	NS	
TIBC (µg/l)	Normal	2787 ± 158	2798 ± 137	2785 ± 144	2789 ± 166	NS	NS	< 0.01
	Overload	3145 ± 177c	3254 ± 175C	3251 ± 169	3195± 166C	NS	NS	
Transferrin saturation (%)	Normal	45.98 ± 0.91	45.32 ± 0.89	46.65± 0.76	46.37± 0.94	NS	NS	< 0.01
	Overload	47.76± 1.32c	47.88± 1.02C	49.59± 0.98c	48.96± 1.05C	NS	NS	
Serum ferritin (µg/l)	Normal	83.25 ± 1.75	82.97 ± 1.68	84.33 ± 1.77	82.34± 1.82	NS	NS	< 0.01
	Overload	87.73 ± 1.87c	86.87± 1.91C	87.91± 1.88c	86.65± 1.96C	NS	NS	
Serum hepcidin (ng/mL)	Normal	14.25 ± 0.59a	14.42± 0.49A	16.85± 0.55b	16.66 ± 0.59B	< 0.01	NS	NS
	Overload	15.75 ± 0.62ac	14.98± 0.58A	17.01 ± 0.61b	16.73± 0.63B	< 0.01	NS	

Values are means ± SEM. (n=10) [1]NS, not significant.
[a,b] Mean values among groups of controls rats fed with different diet and different lower case letters in the same row indicated significant difference by 2-way ANOVA (Tukey's test).
[A,B] Mean values among groups of anemic rats fed with different diet and different upper case letters in the same row indicated significant difference by 2-way ANOVA (Tukey's test).
[c] Mean values of controls rats were significantly different from the corresponding group of rats fed with normal Fe content at $p < 0.05$ by Student's t test.
[C] Mean values of anaemic rats were significantly different from the corresponding group of rats fed with normal Fe content at $p < 0.05$ by Student's t test.
